# Three‐dimensional lower limb alignment in healthy Chinese adults: Gender‐specific phenotypes and ethnic variations for personalised correction

**DOI:** 10.1002/jeo2.70706

**Published:** 2026-04-14

**Authors:** Cheng Liang, Feifan Xiang, Yali Zhang, Lin Liu, Xiaogang Zhang, Lin Qiu, Zhong Li, Yue Chen, Ke Duan, Zhongmin Jin

**Affiliations:** ^1^ School of Mechanical Engineering, Tribology Research Institute Southwest Jiaotong University Chengdu China; ^2^ Department of Orthopedics The Affiliated Hospital of Southwest Medical University LuZhou China; ^3^ Sichuan Provincial Laboratory of Orthopaedic Engineering LuZhou China; ^4^ Department of Nuclear Medicine The Affiliated Hospital of Southwest Medical University LuZhou China; ^5^ School of Mechanical Engineering University of Leeds Leeds UK

**Keywords:** Chinese population characteristics, knee arthroplasty, lower limb alignment, personalised surgery, three‑dimensional classification

## Abstract

**Purpose:**

To develop a personalised three‐dimensional (3D) lower‐limb alignment classification system from a healthy Chinese adult population. Based on full‐length computed tomography (CT) scans, key alignment parameters in the coronal, sagittal and transverse planes were analysed to establish gender‐specific anatomical phenotypes. This system is designed to provide a normative reference for individualised planning in lower‐limb alignment correction procedures such as total knee arthroplasty (TKA) and high tibial osteotomy (HTO).

**Methods:**

Full‐length lower‐limb CT scans were obtained from 554 healthy participants (males: 252, females: 302; age: 22–77 years [mean: 47.7 ± 10.6]), and 3D models were reconstructed. Eleven alignment parameters were measured from the models across the sagittal, coronal and transverse planes. Plane‐specific classifications were developed and integrated into a combined 3D phenotype (CSTA).

**Results:**

All parameters except for joint line obliquity angle showed statistically significant gender‐based differences. The classification system comprises nine sagittal plane types (S1–S9), nine coronal plane types (C1–C9) and three transverse plane types (T1–T3). The most frequent 3D phenotype in males was C1S6T1 (Apex distal/Varus—Moderate/Flexion—External rotation, 10.0%), while in females it was C2S5T2 (Apex distal/Neutral—Moderate/neutral—Neutral, 8.6%). Compared with Western populations, distinct anatomical characteristics were observed in Chinese individuals, including greater femoral anteversion, higher tibial torsion, and a more pronounced tendency towards sagittal‐plane flexion.

**Conclusion:**

This study systematically establishes a 3D lower‐limb alignment classification system for healthy Chinese adults, highlighting gender and ethnicity‑specific alignment characteristics. The proposed classification provides an anatomical basis for personalised surgical planning in procedures such as TKA and HTO, and contributes to advancing lower limb alignment correction towards precision and individualised care.

**Level of Evidence:**

NA, basic science studies.

## Graphical abstract

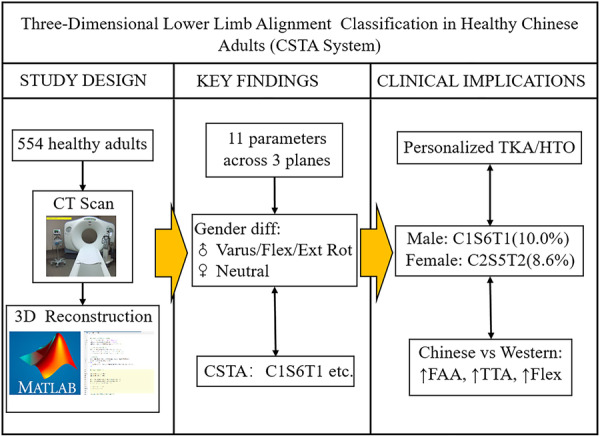

**Abbreviations**
3D: three‐dimensional
AI: artificial intelligence
BMI: body mass index
CPAK: coronal plane alignment classification
CSTA: 3D combined classification
CT: computed tomography
DFO: distal femoral osteotomy
ePDFA: epiphyseal‐based posterior distal femoral angle
FA: functional alignment
FAA: femoral anteversion angle
HKA: hip–knee–ankle angle
HTO: high tibial osteotomy
ICC: intraclass correlation coefficient
JLOA: joint line obliquity angle
KA: kinematic alignment
LDFA: lateral distal femoral angle
MA: mechanical alignment
ML: machine learning
MPTA: medial proximal tibial angle
PDFA: posterior distal femoral angle
PPTA: posterior proximal tibial angle
rKA: restricted kinematic alignment
SD: standard deviation
SJLA: sagittal joint line angle
SMMA: sagittal mechanical medial angle
SPAK: sagittal plane alignment classification
TFI: tibiofemoral index
TKA: total knee arthroplasty
TPAK: transverse plane alignment classification
TTA: tibial torsion angle
UKA: unicompartmental knee arthroplasty

## INTRODUCTION

Lower limb alignment correction procedures, including high tibial osteotomy (HTO) [35], distal femoral osteotomy (DFO) [34], total knee arthroplasty (TKA) [7] and unicompartmental knee arthroplasty (UKA) [23], all rely on accurate lower limb alignment as a surgical guide [41]. With the advancement of personalised medicine, traditional uniform alignment standards have become inadequate in accommodating the anatomical variations among individuals [4]. For instance, mechanical alignment (MA), long regarded as the ‘gold standard’ for TKA, still results in approximately 20% of patients expressing dissatisfaction with postoperative outcomes [31]. One significant reason for this is the neglect of individual alignment specificity. This has driven the evolution of TKA alignment techniques towards personalisation [19, 33], leading to the emergence of various alignment strategies such as kinematic alignment (KA) [32], restricted kinematic alignment (rKA) [42] and functional alignment (FA) [9]. These approaches aim to restore the patient's native knee alignment characteristics, thereby improving satisfaction while preserving function. Concurrently, research on lower limb alignment classification has progressively deepened [2], encompassing classification systems across multiple dimensions, including the coronal plane [22], sagittal plane [27] and transverse plane.

However, for patients requiring knee arthroplasty, severe bilateral knee deformity often makes it difficult to infer their original normal alignment features. Consequently, establishing a personalised alignment standard for surgery becomes challenging, significantly increasing the technical difficulty of the procedure [10]. Therefore, what constitutes the most suitable lower limb alignment for a specific patient, and on what standards it should be based, remain subjects of ongoing debate. Current studies predominantly focus on the classification of arthritis patients [3], lacking systematic analysis of three‐dimensional (3D) alignment characteristics in healthy populations [26]. Establishing a lower limb alignment classification system for healthy individuals holds important clinical significance for providing objective and personalised reference standards for lower limb alignment correction surgeries such as HTO, TKA and UKA.

While the evolution towards personalised alignment strategies (e.g., KA, rKA, FA) is clear, a critical gap remains in preoperative planning: the lack of a detailed, population‐specific reference for normal, 3D lower limb anatomy. Most existing classification systems, including the thorough Coronal Plane Alignment of the Knee (CPAK) classification [15, 22], are derived from two‐dimensional radiographs, which are limited in capturing the complex 3D and rotational alignments of the lower limb. Recent research underscores the value of 3D computed tomography (CT) data in defining a spectrum of native knee phenotypes for surgical planning, highlighting a trend towards data‐driven, individualised surgery [24]. Furthermore, clinical studies indicate that personalised alignment combined with patient‐specific instrumentation can lead to superior functional outcomes compared to traditional methods [12].

Therefore, this gap was addressed in the present study by establishing a comprehensive 3D classification system (CSTA) derived from the normative anatomical data of 554 healthy Chinese adults. The primary aims of this study were defined as follows: (1) establish a normative 3D alignment database for healthy Chinese adults; (2) develop a coupled 3D phenotypic classification system (CSTA); and (3) identify gender‐specific and ethnic‐specific alignment characteristics [6]. It was hypothesised that healthy Chinese adults exhibit distinct 3D alignment phenotypes compared to Western populations, with significant gender‐based variations particularly in rotational and sagittal parameters. By quantitatively defining the coupled variations of 11 key parameters across all three anatomical planes, an effective reference framework is provided by the CSTA system. It enables surgeons to move beyond generic alignment targets towards restoring patient‐specific, prearthritic limb alignment—a cornerstone of contemporary personalised TKA [14]. This normative database is essential for accurate preoperative planning in robotic‐assisted or patient‐specific TKA, HTO and other alignment correction procedures, ultimately aiming to improve implant positioning, soft‐tissue balance and clinical satisfaction.

## MATERIALS AND METHODS

Spatial alignment of the lower limb was analysed in the coronal (for varus/valgus), sagittal (for flexion/extension) and transverse planes (for rotations). This study was based on a CSTA system designed to integrates quantified parameters from all three planes into a composite phenotypic label (‘CxSyTz’). This ‘spatial feature’ aimed to provide a comprehensive anatomical basis for personalised surgical planning.

**Figure 1:**
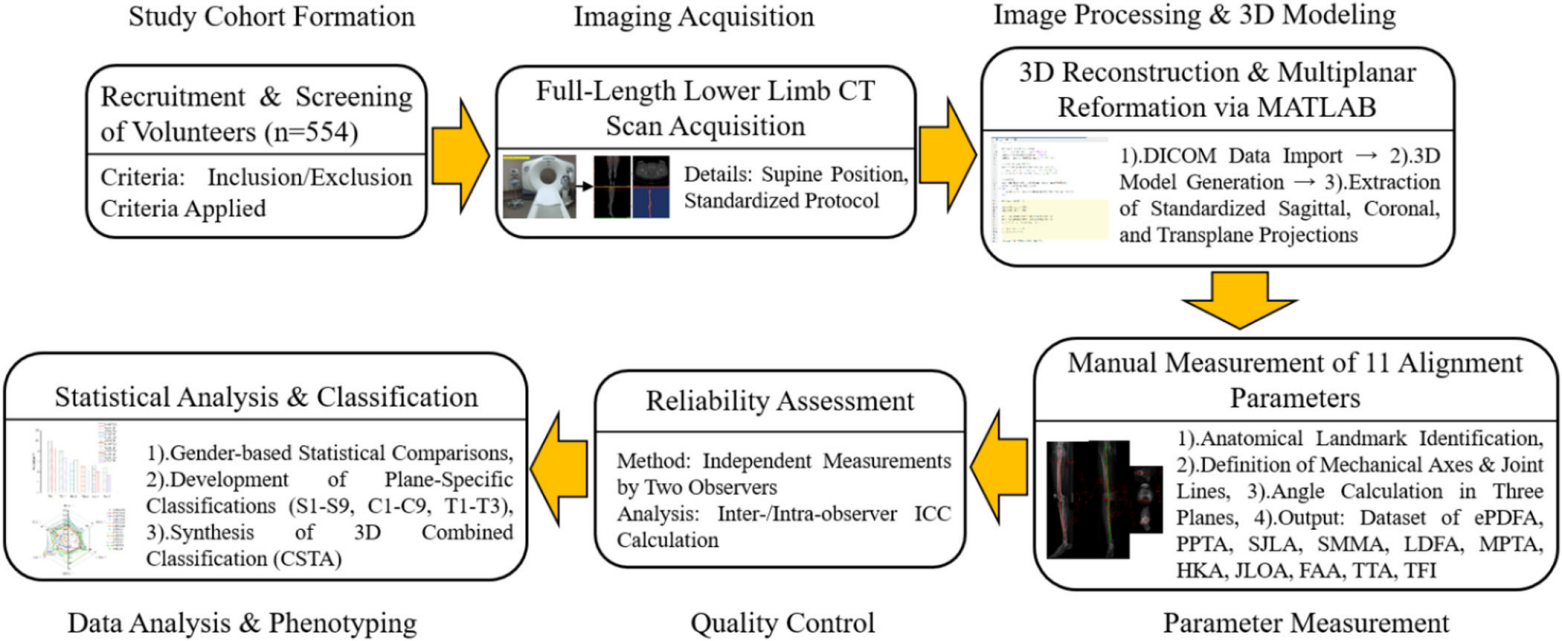
Flowchart illustrating the design of this study. 3D, three‐dimensional; CT, computed tomography; ePDFA, epiphyseal‐based posterior distal femoral angle; FAA, femoral anteversion angle; HKA, hip–knee–ankle angle; ICC, intraclass correlation coefficient; JLOA, joint line obliquity angle; LDFA, lateral distal femoral angle; MPTA, medial proximal tibial angle; PPTA, posterior proximal tibial angle; SJLA, sagittal joint line angle; SMMA, sagittal mechanical medial angle; TFI, tibiofemoral index; TTA, tibial torsion angle.

Accordingly, this study aimed to establish a CSTA system for lower‐limb alignment within a healthy Chinese cohort. Utilising full‐length CT scans from 554 volunteers, 11 key anatomical parameters across the coronal, sagittal and transverse planes were systematically quantified. By statistically analysing coupled variations among these parameters, this work defined a population‐specific reference framework. This framework was designed to provide an anatomical basis for personalising alignment targets in corrective surgeries such as HTO and TKA, as illustrated in Figure 1.

### Ethical approval, subject selection and imaging acquisition

This study was approved by the Ethics Committee of the Affiliated Hospital of Southwest Medical University (Approval No.: KY2024096) and was conducted in accordance with the ethical principles for human research and the Declaration of Helsinki. Healthy adult volunteers were recruited through community advertising. All participants were required to have a normal gait. Participants presenting with any of these conditions were excluded: (1) clinically diagnosed arthritis (including osteoarthritis and rheumatoid arthritis); (2) previous lower limb orthopaedic injury or fracture; (3) congenital or acquired deformity, (4) history of surgery or trauma affecting the lower limb bones or joints, (5) visible malalignment such as knee varus/valgus exceeding 10°, (6) neurological or musculoskeletal conditions affecting gait, (7) systemic inflammatory joint disease, and (8) BMI ≥ 35 kg/m². Consequently, the included participants covered a broad adult age range to capture alignment variation across adulthood, with no specific age subgroup targeted a priori.

All volunteers underwent full‐length lower‐limb scanning using a GE LightSpeed VCT 64‐slice spiral CT scanner. The scanning parameters were set as follows: slice thickness 1 mm, in‐plane resolution 0.625 × 0.625 mm. Participants were positioned in a standard supine posture with lower limbs adducted and toes pointing forward to ensure consistency of positioning [28, 29]. Basic participant information is summarised in Table 1.

**Table 1:** Demographic characteristics of the volunteers.

Variable	Male (*n* = 252) (M ± SD, MAX, MIN)	Female (*n* = 302) (M ± SD, MAX, MIN)	*p*‐value
Age (year)	47.9 ± 12.4, 71.0, 24.0	47.4 ± 8.8, 67.0, 22.0	0.60^a^
BMI (kg/m^2^)	23.9 ± 3.3, 31.2, 16.0	23.8 ± 3.2, 32.8, 15.8	0.41^a^

Abbreviations: BMI, body mass index; SD, standard deviation.

^a^ Independent‐samples *t*‐test.

### Imaging data processing and measurement

**Figure 2:**
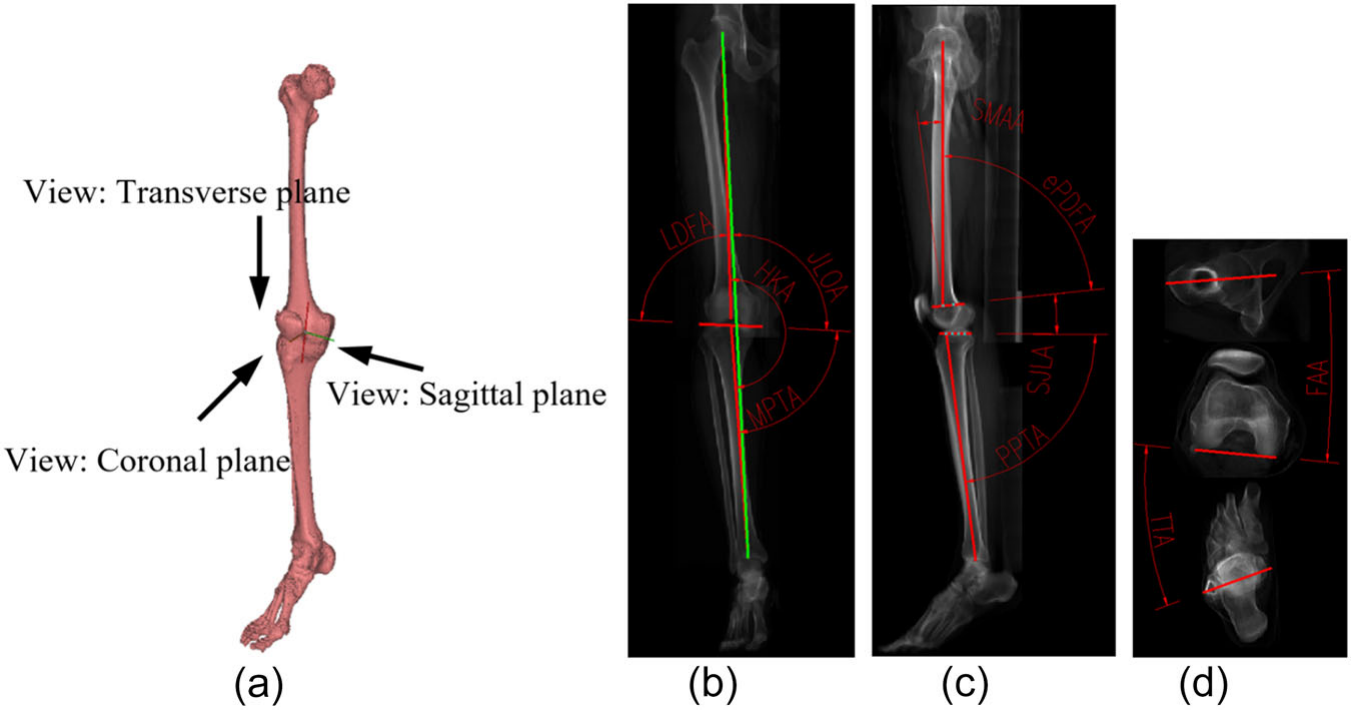
Measurement diagrams of 11 alignment parameters. (a) Schematic diagram of the selection of sagittal, coronal and transverse perspectives. (b) Schematic diagram of coronal plane parameter measurement. (c) Schematic diagram of sagittal plane parameter measurement. (d) Schematic diagram of transverse plane parameter measurement.

Acquired DICOM‐format CT data were imported into MATLAB 2021 (MathWorks) for 3D reconstruction and analysis. Projection images of both lower limbs in the sagittal, coronal and transverse planes were extracted separately, as illustrated in Figure 2a. Measurement parameters from each plane and their corresponding anatomical landmark definitions are described below:

1. Coronal plane parameters (Figure 2b)
  Based on related research [18, 38], the following parameters were defined:
  a. Lateral distal femoral angle (LDFA): the lateral angle between the femoral coronal mechanical axis and the joint line.
  b. Medial proximal tibial angle (MPTA): the medial angle between the tibial coronal mechanical axis and the joint line.
  c. Hip–knee–ankle angle (HKA): the medial angle between the femoral coronal mechanical axis and the tibial coronal mechanical axis.
  d. Joint line obliquity angle (JLOA): the proximal medial angle between the joint line and the lower limb coronal mechanical axis.

Here, the femoral mechanical axis was defined as the line connecting the centre of the femoral head and the centre of the knee joint; the tibial mechanical axis as the line connecting the centre of the knee joint and the centre of the ankle joint; and the lower limb mechanical axis as the line connecting the centre of the femoral head and the centre of the ankle joint.

2. Sagittal plane parameters (Figure 2c)
  With reference to established methods [39], the following parameters were measured:
  a. Epiphyseal‐based posterior distal femoral angle (ePDFA): the posterior angle between the distal femoral joint line and the femoral sagittal mechanical axis.
  b. Posterior proximal tibial angle (PPTA): the posterior angle between the proximal tibial joint line and the tibial sagittal mechanical axis.
  c. Sagittal joint line angle (SJLA): the angle between the distal femoral joint line and the proximal tibial joint line.
  d. Sagittal mechanical medial angle (SMMA): the angle between the femoral sagittal mechanical axis and the tibial sagittal mechanical axis.

In the sagittal plane, joint lines were defined based on consistent bony landmarks: the distal femoral joint line determined by connecting the anterior and posterior margins of the distal femoral epiphyseal line (physeal scar), while the proximal tibial joint line was defined by the bony margins of the tibial plateau [1]. The femoral sagittal mechanical axis was defined by connecting the centre of the femoral head to the anterior one‐third point of the defined distal femoral joint line, and the tibial sagittal mechanical axis extended from the anterior one‐fifth point of the proximal tibial joint line to the centre of the ankle joint. These proportional landmarks were selected based on established deformity correction planning standards to ensure reproducibility and minimise variability associated with anterior cortical line definitions [1, 39]. The epiphyseal line was selected over the articular surface to optimise measurement reproducibility due to the superior clarity on CT and to provide a reference reflecting inherent bony morphology, independent of articular cartilage variation [5]. Correspondingly, the measured angle was termed ePDFA to distinguish it from the conventional PDFA. PPTA and SJLA were derived accordingly. SMMA, calculated as the angle between the femoral and tibial mechanical axes, served as a composite indicator of overall limb posture, where a positive value indicates a relative flexion alignment.

Although the SJLA is conceptually defined at the knee's maximum extension, it is acknowledged that in the standardised supine, non‐weight‐bearing position adopted for CT acquisition, the knee may not achieve the same degree of active maximal extension as in a standing or stressed examination. However, this standardised relaxed position was chosen to ensure consistency across all participants and to minimise positional variability. The SJLA measured in this study thus represents the passive, relaxed alignment of the joint in a reproducible clinical imaging scenario, which remains a valid reference for establishing population norms and for preoperative planning in similarly positioned imaging.

3. Transverse plane parameters (Figure 2d)
  Following established methods [13, 30, 37], the following were measured:
  a. Femoral anteversion angle (FAA): the angle between the femoral neck axis and the posterior condylar axis of the femur in the transverse plane.
  b. Tibial torsion angle (TTA): the angle between the transmalleolar axis and the posterior condylar axis of the femur in the transverse plane.
  c. Tibiofemoral index (TFI): defined as the difference between TTA and FAA (positive values indicate external rotation, negative values indicate internal rotation).

To ensure data reliability and minimise measurement error, a rigorous protocol was implemented. Standardised training was completed by both investigators to establish consensus on anatomical landmarks and procedures. Each parameter was measured twice per observer, with discrepancies exceeding a predefined threshold triggering a third measurement; the two closest values were then averaged. The inter and intraobserver reliability for all parameters were quantitatively assessed using the intraclass correlation coefficient (ICC) based on a two‐way random‐effects model [20], with ICC > 0.75 indicating excellent reliability. The final values used for all analyses and classification were the averages derived from the two observers’ measurements.

### Statistical analysis

Data were compared by *t*‐tests (Prism 8.02, GraphPad), and a *p* < 0.05 was considered statistically significant.

## RESULTS

### Comparison of 3D measurement parameters

A total of 554 healthy knee datasets were included (252 males, 302 females). No statistically significant differences in age or body mass index (BMI) were observed between the two groups (*p* > 0.05), indicating comparability and suitability for subsequent classification analysis. Basic participant information is presented in Table 1.

Gender‐based comparisons of the measurement parameters across all planes are summarised in Table 2. Except for JLOA, all remaining parameters showed statistically significant differences between males and females (*p* < 0.05), suggesting that gender is an important factor influencing lower limb alignment characteristics.

**Table 2:** Gender comparison results of the measured parameters.

Variable	Male/(M ± SD, MAX, MIN)	Female/(M ± SD, MAX, MIN)	*p*‐value
ePDFA	77.6 ± 2.8, 84.0, 70.5	78.5 ± 3.0, 85.8, 71.4	0.000^a^^,^ *
PPTA	84.7 ± 1.9, 89.8, 77.5	84.3 ± 1.9, 88.7, 78.6	0.029^a^^,^ *
SJLA	13.8 ± 3.7, 23.6, 6.7	14.8 ± 4.2, 23.1, 6.9	0.001^a^^,^ *
SMMA	4.0 ± 2.5, 10.7, −2.6	2.4 ± 3.5, 11.8, −5.4	0.000^a^^,^ *
LDFA	86.8 ± 2.3, 92.6, 80.3	86.4 ± 2.3, 92.9, 80.6	0.038^a^^,^ *
MPTA	84.6 ± 2.5, 90.7, 77.8	85.6 ± 2.4, 91.7, 78.9	0.000^a^^,^ *
HKA	177.8 ± 2.3, 182.7, 172.0	179.1 ± 2.2, 185.4, 170.9	0.000^a^^,^ *
JLOA	94.2 ± 2.1, 101.4, 88.8	94.0 ± 2.1, 99.8, 88.2	0.242^a^
FAA	18.9 ± 8.1, 43.4, −3.9	24.9 ± 8.2, 47.1, 0.5	0.000^a^^,^ *
TTA	30.7 ± 8.2, 49.8, 9.1	34.4 ± 7.8, 49.6, 11.2	0.000^a^^,^ *
TFI	11.8 ± 10.0, 34.7, −16.8	9.6 ± 10.6, 33.9, −19	0.012^a^^,^ *

Abbreviations: ePDFA, epiphyseal‐based posterior distal femoral angle; FAA, femoral anteversion angle; HKA, hip–knee–ankle angle; JLOA, joint line obliquity angle; LDFA, lateral distal femoral angle; MPTA, medial proximal tibial angle; PPTA, posterior proximal tibial angle; SD, standard deviation; SJLA, sagittal joint line angle; SMMA, sagittal mechanical medial angle; TFI, tibiofemoral index; TTA, tibial torsion angle.

a Independent‐samples *t*‐test.

* Statistically significant.

### Establishment of the 3D classification system

**Figure 3:**
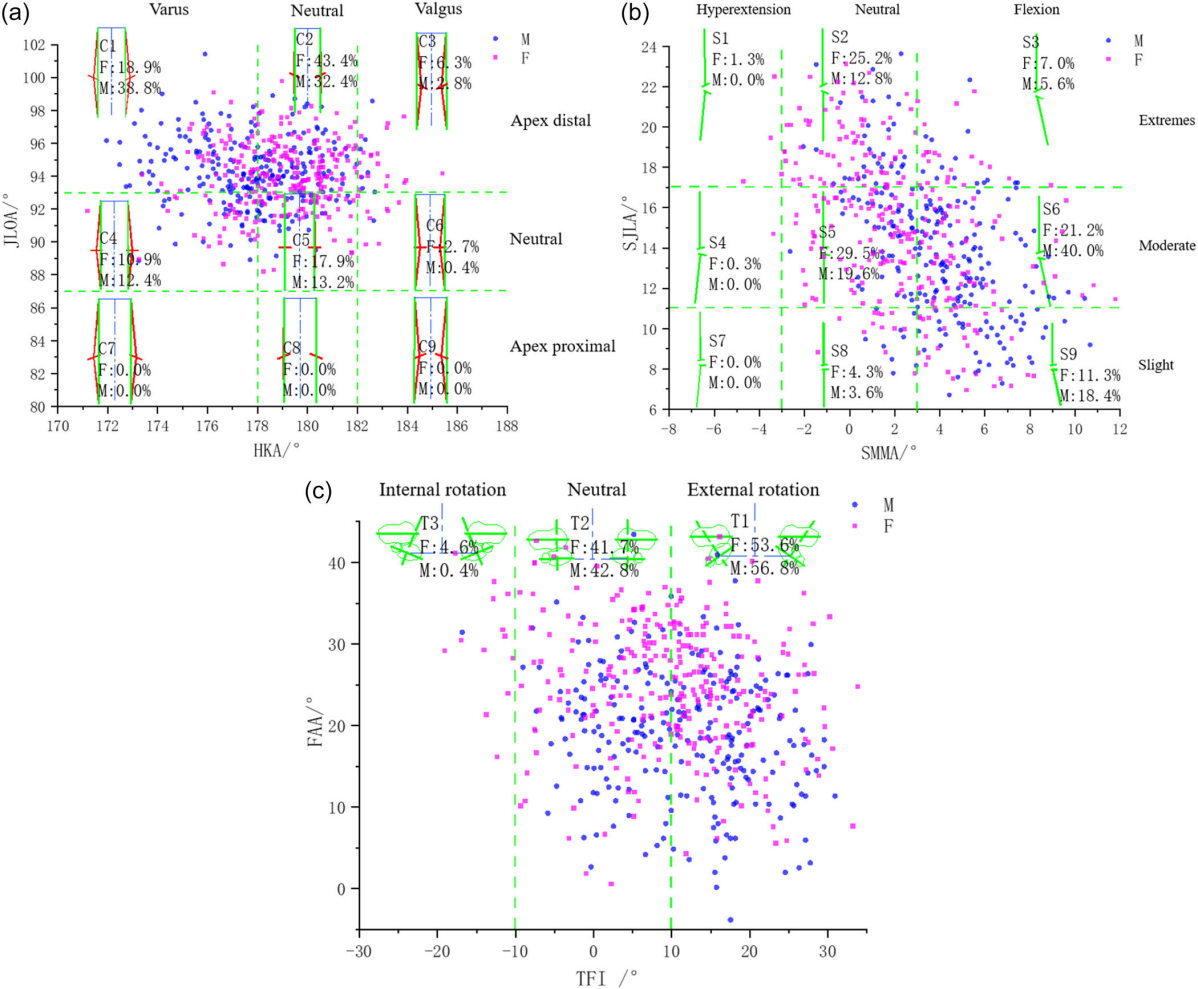
Distribution of lower‐limb alignment types within the healthy Chinese cohort for each plane. (a) CPAK distribution: Showing the prevalence of CPAK types (C1–C9) in males and females. Males predominantly exhibited varus types (C1), while females showed a higher frequency of neutral alignment (C2). Types C7–C9 were not observed. (b) SPAK distribution: Showing the prevalence of SPAK types (S1–S9). The most common type in males was S6 (indicating a flexion tendency), whereas S5 (neutral) was most frequent in females. (c) TPAK distribution: Showing the prevalence of TPAK types (T1–T3). Both genders were predominantly T1 (external rotation) or T2 (neutral), with T3 (internal rotation) being rare. Females exhibited a significantly higher proportion of high FAA. CPAK, coronal plane alignment classification; FAA, femoral anteversion angle; HKA, hip–knee–ankle angle; SMMA, sagittal mechanical medial angle; SPAK, sagittal plane alignment classification; TFI, tibiofemoral index; TPAK, transverse plane alignment classification.

**Figure 4:**
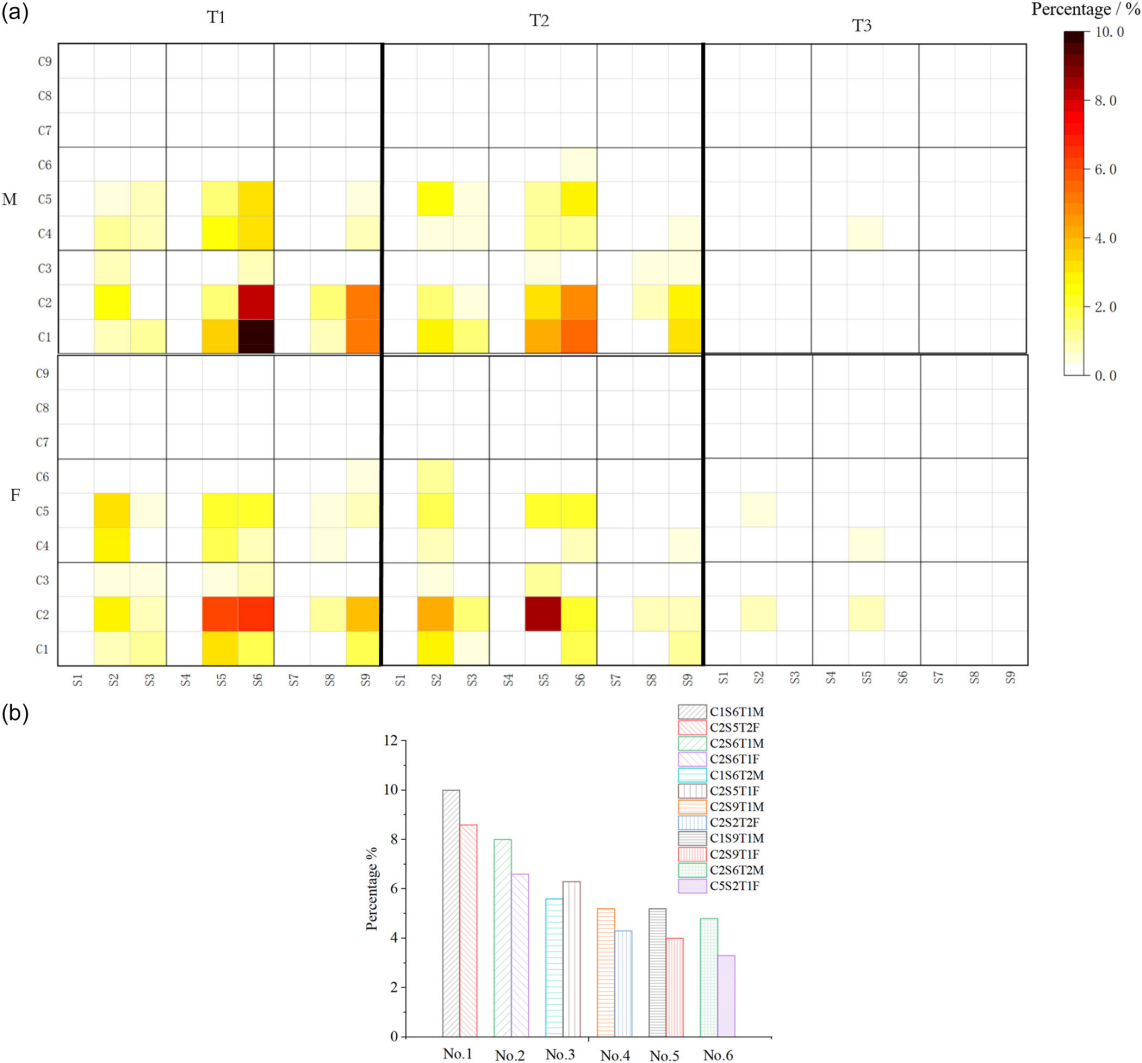
3D coupled Classification (CSTA): Integration and distribution of multi‐plane phenotypes. (a) Heatmap of CSTA phenotype distribution: A 3D matrix visualising the frequency of each combined ‘CxSyTz’ phenotype across the cohort. Warmer colours indicate higher prevalence, highlighting the most common integrated alignment profiles (e.g., C1S6T1 in males, C2S5T2 in females). (b) Ranking of the top CSTA phenotypes: Bar chart displaying the six most frequent CSTA types for males and females separately.

To establish clinically meaningful categories from the continuum of anatomical variation, the classification boundaries for sagittal, coronal and transverse plane phenotypes were defined based on the distribution characteristics—specifically, the standard deviation (SD)—of their respective alignment parameters within the healthy cohort. Following the methodological principles of established coronal plane classifications [22], thresholds for key composite parameters were set at intervals of approximately one SD from the overall sample mean. This statistically driven process resulted in distinct plane‐specific classification systems, as shown in Figure 3. These individual systems were subsequently integrated into forming the CSTA (Figure 4).

1. Coronal plane classification (C1–C9):
  Based on LDFA, MPTA, HKA and JLOA. This classification describes varus/valgus alignment and joint line obliquity of the lower limb.
2. Sagittal plane classification (S1–S9):
  Based on ePDFA, PPTA, SJLA and SMMA. This classification reflects the flexion‐extension posture of the knee in the sagittal plane.
3. Transverse plane classification (T1–T3):
  Based on FAA, TTA and TFI. This classification characterises the rotational relationship between the femur and tibia.
4. The CSTA is expressed in the format ‘CxSyTz’ (e.g., C1S6T1), forming an individualised ‘lower limb spatial phenotype’ that reflects intersubject variation.

### Distribution characteristics of plane‐specific classifications

Based on the distribution patterns of anatomic parameters (Figure 3), the following characteristics were identified for each plane.

#### CPAK

Type C1 was predominant among male participants, whereas type C2 was most common among female ones (Figure 3a). In males, the most frequent types were: C1 (38.5%), C2 (32.5%), C5 (13.5%), C4 (12.3%), C3 (2.8%) and C6 (0.4%). In females, the order was: C2 (43.4%), C1 (18.2%), C5 (18.2%), C4 (11.2%), C3 (6.3%) and C6 (2.7%). Types C7, C8 and C9 were not observed. Overall, males tended towards varus alignment, while females were closer to neutral alignment.

#### Sagittal plane alignment classification (SPAK)

Type S6 was predominant in the males, whereas type S5 was most common in the females (Figure 3b). In the males, the most frequent types were: S6 (40.0%), S5 (19.6%), S9 (18.4%), S2 (12.8%), S3 (5.6%) and S8 (3.6%). In the females, the order was: S5 (29.5%), S2 (25.2%), S6 (21.2%), S9 (11.3%), S3 (7.0%) and S8 (4.3%). Types S1, S3, S4, S7 and S8 were rare in these healthy populations. Overall, males exhibited a greater tendency towards flexion, while females tended towards larger joint angles.

#### Transverse plane alignment classification (TPAK) distribution

Both the males and females were predominantly classified as T1 (external rotation) or T2 (neutral rotation), with T3 (internal rotation) being rare (Figure 3c). In the males, T1 accounted for 56.8% and T2 for 42.8%. In the females, T1 accounted for 53.6% and T2 for 41.7%. Females exhibited significantly greater FAA than males.

### Distribution of CSTA and establishment of radar chart

Common types of CSTA showed gender‐specific differences (Figure 4a). The most frequent type in males was C1S6T1 (10.0%, Apex distal/Varus—Moderate/Flexion—External rotation), followed by C2S6T1 (8.0%, Apex distal/Neutral—Moderate/Flexion—External rotation). In females, the most frequent types were C2S5T2 (8.6%, Apex distal/Neutral—Moderate/neutral—Neutral) and C2S6T1 (6.6%, Apex distal/Neutral—Moderate/Flexion—External rotation). The top six 3D phenotypes in healthy males were: C1S6T1 (10.0%), C2S6T1 (8.0%), C1S6T2 (5.6%), C2S9T1 (5.2%), C1S9T1 (5.2%) and C2S6T2 (4.8%). In females, the order was: C2S5T2 (8.6%), C2S6T1 (6.6%), C2S5T1 (6.3%), C2S2T2 (4.3%), C2S9T1 (4.0%) and C5S2T1 (3.3%) (Figure 4b).

Overall, females exhibited 3D lower limb alignment that was closer to neutral compared to males, whereas males tended towards varus in the coronal plane, flexion in the sagittal plane and external rotation in the transverse plane.

The predominance of specific CSTA types suggests distinct, gender‐associated alignment patterns. For instance, the high frequency of phenotypes with coronal varus (C1), sagittal flexion (S6) and external rotation (T1) in males may indicate a constitutional alignment that predisposes to specific load distributions and biomechanical environments. Conversely, the common female phenotype of coronal neutrality (C2) and sagittal neutrality (S5) aligns with different functional demands. These CSTA ‘profiles’ establish a normative benchmark; deviation from them in arthritic knees may semi‐quantitatively sign disease‐related deformity and inform the goal of ‘phenotype restoration’ in personalised surgery.

**Figure 5:**
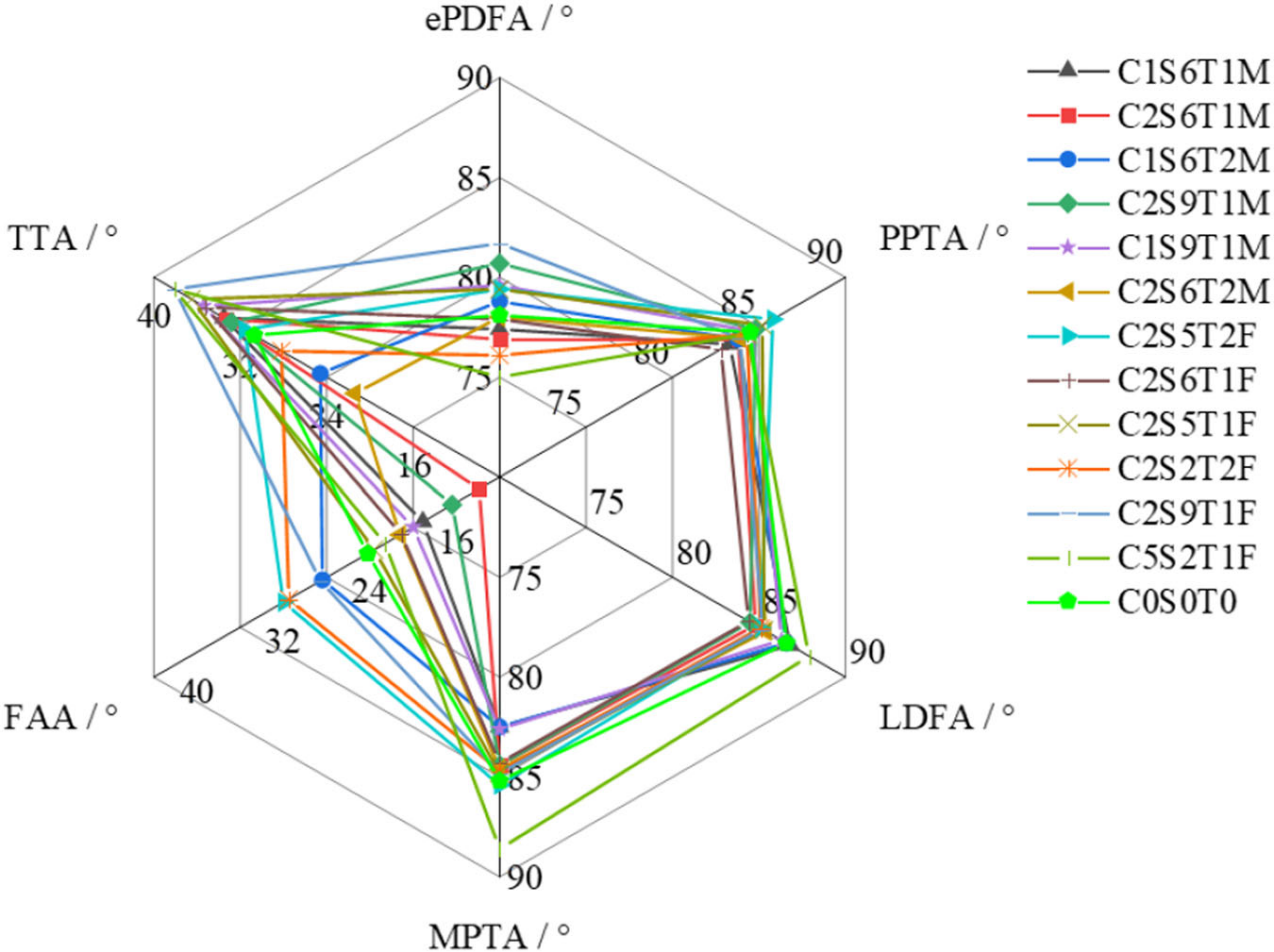
Radar chart visualisation of the six most common CSTA phenotypes for males and females. Each axis represents one of the 11 normalised 2.2 alignment parameters. The line (‘C0S0T0’) represents the mean values of the entire cohort as a reference. ePDFA, epiphyseal‐based posterior distal femoral angle; FAA, femoral anteversion angle; LDFA, lateral distal femoral angle; MPTA, medial proximal tibial angle; PPTA, posterior proximal tibial angle; TTA, tibial torsion angle.

Based on the 3D classification results, a radar chart was created (Figure 5) using each parameter as a dimension to display the top six classification curves for male and female groups, with the overall mean curve of the mixed male‐female sample (C0S0T0) serving as the reference baseline. Analysis revealed that TTA appeared as a prominent dimension in most classifications (curves mostly located outside the mean), while ePDFA, PPTA and FAA were generally distributed around the mean (curves evenly appearing inside and outside the mean). LDFA and MPTA frequently appeared as recessed dimensions (curves mostly located inside the mean).

## DISCUSSION

In the present study, a 3D classification system was established for lower limb alignment based on full‐length CT scans from 554 healthy Chinese adults. The primary findings are threefold. First, a comprehensive phenotypic classification encompassing the coronal (C1–C9), sagittal (S1–S9) and transverse (T1–T3) planes was developed, integrating them into a combined ‘CxSyTz’ format. Second, significant gender‐based differences were identified across almost all parameters, with males tending towards varus, flexion and external rotation phenotypes, whereas females demonstrated alignment closer to neutral. Third, compared to Western populations, these Chinese adults exhibited distinct anatomical characteristics, including greater femoral anteversion, higher tibial torsion and a reduced SJLA. These findings are interpreted in the following discussion in relation to existing literature, exploring their clinical implications and addressing limitations of the present study.

### Systematic comparison and clinical implications

#### CPAK

The coronal plane classification established in this study aligns closely in principle with the CPAK classification proposed by MacDessi et al., as both systems utilise two independent variables (i.e., HKA, JLOA) for phenotypic categorisation [22]. In this study, the data further substantiated the applicability of the CPAK framework within a healthy Chinese population by providing specific parameter distributions. The mean HKA observed in the Chinese males was 177.8° ± 2.3°, indicating a mild varus tendency, consistent with earlier reports of a higher prevalence of constitutional varus in Asian populations [17, 40]. In contrast, the mean HKA in females (179.1° ± 2.2°) was closer to neutral alignment. Although the LDFA and MPTA measured from this population were approximately 1° lower than values reported in some other studies [22], their trends remained consistent. This structural relationship may underline the lack of significant gender difference observed in JLOA, supporting the notion that ‘JLOA is independent of gender’ [21]. These findings hold direct clinical relevance: during TKA for Asian patients with constitutional varus, strictly targeting an absolutely neutral mechanical axis (0°) may not represent the optimal objective. Personalised alignment strategies, such as rKA, may offer a more physiological approach to restoring native joint biomechanics and soft‐tissue balance.

#### SPAK

In the sagittal plane, SJLA measured in this study (males: 13.8° ± 3.7°; females: 14.8° ± 4.2°) were lower than the reference values of approximately 16° reported for Western populations [1]. Concurrently, ePDFA was also lower in this Chinese population (males: 77.6° ± 2.8°; females: 78.5° ± 3.0°) compared with Western counterparts (81.1° ± 3.9°) [1, 39], whereas PPTA (approximately 84.7°) was similar. This indicates that while different ethnic groups share similar anatomical structures for maintaining tibial plateau stability, morphological variations in the distal femur may be a key factor influencing knee flexion function. Geometrically, SJLA, along with SMMA, reflects the interrelation between skeletal morphology and soft‐tissue influence: an increase in SJLA is typically associated with a decrease in SMMA, presenting as a neutral or hyperextended posture, whereas a decrease in SJLA results in an increased SMMA, indicative of a flexed posture. It should be noted that the SJLA in this study was derived from standardised supine CT, captured under a passive, relaxed state; without concomitant weight‐bearing or active motion assessment, its numerical value should be interpreted as a reproducible anatomical reference under these conditions rather than a direct quantitative measure of clinical soft‐tissue contracture. Additionally, the definition of a ‘neutral’ sagittal phenotype (e.g., S5) in the classification is statistically derived from the distribution within this healthy population, describing the most common pattern without asserting a universal physiological benchmark. Consequently, when assessing knee flexion contracture or planning sagittal balance in TKA, these ethnic‐specific normative values should be considered.

#### TPAK

The data quantified distinct rotational profiles in the Chinese cohort: the mean FAA was 18.9° ± 8.1° in males and 24.9° ± 8.2° in females, both exceeding the reference range of approximately 10°–22° reported for Western populations [5]. The significantly greater FAA in females aligns with trends observed in Western studies [11]. Regarding tibial torsion, TTA was 30.7° ± 8.2° in males and 34.4° ± 7.8° in females, which are higher than the typical Western range of 20°–30° [8], with a notable gender difference. Consequently, TFI was 11.8° ± 10.0° in Chinese males, indicating a more pronounced lower limb external rotation pattern compared to the commonly reported <10° in Western groups [25]. These ethnic anatomical characteristics have clear technical implications for knee surgery, particularly TKA. Greater FAA may necessitate adjusting the external rotation of the femoral component to mitigate patellofemoral tracking issues, while increased tibial external rotation can influence tibial component positioning and the patellar tendon‐prosthesis relationship. Furthermore, the markedly higher FAA in females suggests a relatively weaker medial constraint mechanism for the patella, potentially elevating the risk of patellofemoral instability. Therefore, the inherently greater bony torsion in Asian patients, especially gender‐specific differences, warrants specific consideration during preoperative planning and implant selection.

#### CSTA and the radar chart

The integration of the three‐plane classifications into the CSTA system provides an individualised ‘spatial map’ of lower limb alignment. The most frequent phenotypes differed between genders (C1S6T1 in males, C2S5T2 in females), offering quantitative evidence for understanding gender‐associated alignment patterns. Although derived from a healthy population, the detailed 3D alignment reference database established herein forms a foundation for future research into the relationship between joint degeneration, deformity progression and native anatomical phenotype. It provides a spectrum of specific, population‐based anatomical targets for the personalised surgical philosophy of ‘restoring the patient's native alignment’.

Further observation of the connection between LDFA and MPTA in the radar chart shows that when this line is parallel to the reference contour, the corresponding HKA approaches 180°. Since LDFA is generally greater than MPTA, it indicates that in most classifications, the joint line tends to incline inward. Notably, the relationship between LDFA and MPTA essentially reflects the intrinsic connection between HKA and JLOA; that is, analysing LDFA‐MPTA is equivalent to analysing HKA‐JLOA. Similarly, the connection between FAA and TTA can also reflect rotational alignment characteristics: when this line is parallel to the reference contour, TFI approaches 0°. In practice, TTA is often greater than FAA, suggesting that most classifications exhibit overall lower limb external rotation, and the greater the deviation from parallelism, the more pronounced the external rotation tendency. Regarding sagittal plane parameters, if SMMA is assumed to be 0° (i.e., vertical sagittal alignment of the lower limb), SJLA can be calculated by the formula SJLA = 180°—ePDFA—PPTA, meaning the sum of the remaining angles of ePDFA and PPTA dimensions in the radar chart equals SJLA. Therefore, for a comprehensive analysis of sagittal alignment characteristics, selecting any three of the four parameters—ePDFA, PPTA, SJLA and SMMA—is sufficient to fully characterise the morphology of this plane.

In healthy individuals (i.e., without coronal joint line deformation or sagittal soft tissue contracture), the parameter relationships presented in this radar chart are equivalent to the classification features described in Figure 3, further validating the consistency and reliability of this 3D classification system in characterising physiological lower limb alignment.

#### Future directions: Integration with advanced technologies for personalised surgery

The quantitative data and classification framework of the CSTA system constitute a foundational dataset that is highly compatible with emerging digital technologies, thereby laying the groundwork for data‐driven personalised orthopaedics. First, this structured 3D phenotypic database can serve as a critical input for machine learning (ML) and artificial intelligence (AI) algorithms [36]. By correlating preoperative CSTA phenotypes with postoperative outcomes—such as patient‐reported function, implant survivorship, or complication risk—predictive models can be developed to provide decision support for formulating truly personalised surgical plans with potentially superior prognoses [16]. Secondly, the CSTA classification can be directly integrated into the software workflow of robot‐assisted surgery and patient‐specific instrumentation. Planning software could automatically identify a patient's specific ‘CxSyTz’ phenotype and accordingly plan osteotomy extent and soft‐tissue balancing targets, aiming to restore their individualised 3D alignment rather than pursuing a uniform mechanical axis. Finally, the common phenotypic characteristics revealed by such population data—for instance, the high femoral anteversion and tibial torsion observed in this study—can provide crucial evidence for the future anatomical design and dimensional optimisation of knee prostheses. This ensures better fit and biomechanical performance for specific demographic groups. Therefore, the CSTA system is not merely a descriptive tool but may become a key bridge connecting individualised anatomical understanding with precise surgical implementation.

### Interpretation of phenotypic characteristics and pathways for clinical translation

Asian populations, particularly males, exhibited a higher proportion of varus knees. Females generally demonstrated greater femoral anteversion and more pronounced tibial external rotation. Consequently, special attention should be paid to patellar stability and mediolateral load balance during preoperative planning and postoperative rehabilitation. From a biomechanical perspective, it was found that male lower limbs tend towards a varus, flexed and externally rotated alignment pattern, whereas female alignment is closer to neutral. This specific alignment may confer greater joint stability under both static and dynamic loading conditions. This finding aligns with the evolutionary and social roles in which males have historically engaged in more strength‐based activities. The predominant lower‐limb spatial phenotypes in males (e.g., C1S6T1) may indicate a higher predisposition to medial compartment osteoarthritis. In contrast, common female phenotypes (e.g., C2S5T2) might be associated with a higher risk of patellofemoral instability.

The 3D classification system provided a ‘stereoscopic profile’ of lower limb alignment, facilitating more intuitive and systematic communication in clinical teaching, doctor‑patient interactions and surgical planning. A systematic evaluation of the patient's 3D classification is essential before lower limb alignment correction procedures (e.g., HTO, DFO) or joint replacement surgeries (e.g., TKA, UKA). Transverse plane parameters (FAA, TTA) significantly influence patellar tracking, while coronal and sagittal plane parameters are directly related to joint load distribution and soft‑tissue balance. For example, in performing TKA, CPAK should guide varus/valgus release strategies, SPAK should inform adjustments of flexion/extension gaps and TPAK should be referenced to optimise rotational alignment. In patellar surgery, femoral anteversion and tibial external rotation should be comprehensively assessed, and the decision to perform concomitant rotational osteotomy should be based on TFI. Therefore, routine 3D classification assessment is recommended for patients undergoing TKA, lower limb alignment correction, or patellar surgery to guide the development of individualised surgical plans.

### Study limitations and future perspectives

Several limitations should be considered when interpreting the results of this study. First, its cross‐sectional and single‐centre design limited the generalisability of the normative values. While the sample size was medium, all participants were from one geographic region, and future multi‐centre studies across diverse Asian populations are needed to establish more robust reference ranges. Moreover, similar to the CPAK classification methodology [22], the classification thresholds are derived from ±1 SD within this cohort, which may limit external generalizability to populations with different anatomical variances.

Second, constraints are imposed by the imaging protocol itself. All measurements were derived from CT scans obtained in a standard supine, non‐weight‐bearing position. Although this ensures excellent bone visualisation and measurement reproducibility, the obtained alignment parameters, particularly those in the sagittal plane (e.g., SJLA), may not fully reflect the FA under weight‐bearing conditions or active muscle forces. Furthermore, the study did not include a direct assessment of knee range of motion, which limits the interpretation of SJLA as a direct measure of soft‐tissue contracture.

Third, there are inherent methodological considerations. The use of the epiphyseal line to define the distal femoral joint line (resulting in the ePDFA) was chosen for reproducibility. This practice, however, yielded values that differ systematically from the conventional PDFA based on the articular surface. Additionally, the use of proportional anterior landmarks (1/3 and 1/5) for defining sagittal mechanical axes [1], while enhancing reproducibility, may influence angle measurements and classification boundaries compared to alternative definitions. This should be noted when comparing our data with other studies.

Fourth, and most importantly for clinical translation, this study established a normative database from a healthy population. The direct applicability of these CSTA phenotypes to guiding surgical planning for osteoarthritic patients undergoing TKA, HTO, or UKA still needs to be validated. The association between specific 3D phenotypes and risks of osteoarthritis progression, optimal prosthetic positioning, or patient‐reported outcomes remains an essential area for future longitudinal and clinical outcome studies.

Finally, this work presented a static anatomical classification. The dynamic interaction of these 3D parameters during gait or other activities and their integration with emerging technologies (e.g., patient‐specific instrumentation, robotic surgery, artificial intelligence for predictive planning), are beyond the scope of this study but represent interesting future directions.

## CONCLUSION

Based on full‐length lower limb CT data from 554 healthy Chinese adults, a CSTA encompassing the coronal, sagittal and transverse planes was established. Significant gender‐based differences in lower limb alignment parameters were identified, with the males exhibiting a tendency towards varus, flexion and external rotation phenotypes, whereas the females demonstrated alignment closer to neutral. Furthermore, distinct anatomical characteristics, including greater femoral anteversion and higher tibial torsion, were delineated in this Asian population compared to Western counterparts. The CSTA system provides a detailed phenotypic map of physiological lower limb alignment variation. While derived from a healthy population, this normative database establishes a crucial reference framework and offers a potential tool for understanding individual anatomical predispositions. Future research is warranted to correlate these 3D phenotypes with the development of osteoarthritis and to validate their application to preoperative planning for knee reconstructions. Overall, this study provided a basic framework for guiding more individualised approaches in lower limb alignment correction surgery.

## AUTHOR CONTRIBUTIONS

**Cheng Liang**: Methodology; writing—original draft; writing—review and editing. **Feifan Xiang**: Software; resources. **Yali Zhang**: Software; resources. **Lin Liu**: Software; resources. **Xiaogang Zhang**: Revision; resources; writing—review and editing. **Lin Qiu**: Revision; supervision. **Zhong Li**: Methodology; writing—review and editing. **Yue Chen**: Revision; supervision. **Ke Duan**: Methodology; writing—review and editing. **Zhongmin Jin**: Conceptualisation; resources; supervision.

## CONFLICT OF INTEREST STATEMENT

The authors declare no conflicts of interest.

## ETHICS STATEMENT

This study was approved by the Ethics Committee of the Affiliated Hospital of Southwest Medical University, approval number: KY2024096. All procedures were conducted in accordance with the Declaration of Helsinki. Informed consent was obtained from all individual participants included in the study. Clinical trial number: not applicable.

## Data Availability

The data cannot be made publicly available upon publication because they contain sensitive personal information. The data that support the findings of this study are available upon reasonable request from the corresponding authors.
